# A survey on non specialized off-the-shelf JPEG2000 viewers for digital microscopy use

**DOI:** 10.1186/1746-1596-3-S1-S20

**Published:** 2008-07-15

**Authors:** Vincenzo Della Mea, Nicola Bortolotti, Carlo Alberto Beltrami

**Affiliations:** 1 Medical Informatics, Telemedicine and eHealth Lab, Dept. of Maths & Computer Science, University of Udine, 33100 Udine, Italy; 2 Section of Pathology, Dept. of Medical Morphological Research, University of Udine, 33100 Udine, Italy

## Abstract

The present paper will present a survey on features of a number of non-specialized off-the-shelf JPEG2000 viewers, seen from the point of view of digital microscopy. Selected viewers were tested within a number of usage scenarios, including: i) open a conformance test JPEG2000 file; ii) open a large JPEG2000 file; iii) moving from one point to another; iv) changing resolution/magnification. For each scenario, data recorded included: successful or unsuccessful operation; time needed for conclusion; occasional problems.

Preliminary results demonstrate that JPEG2000 conformance as stated by many viewers is only limited to some of the possibilities of the JPEG2000 standard, in particular for what regards file size.

## Introduction

Although standardization for image formats in Pathology is still not completed, much interest has been devoted to JPEG2000 as a format to store digital slides [1]. JPEG 2000 is a wavelet-based image compression standard. The Joint Photographic Experts Group committee created it in the year 2000 with the intention of replacing the JPEG standard. As JPEG2000 is not meant as a specialized format for biomedical images, but is rather devoted to a broad range of applications including geospatial imaging, artistic images, etc, a number of image viewers are available that declare JPEG2000 compatibility.

However, digital slides, even when coded in JPEG2000, are peculiar images as their size is on average much larger than any other kind of images, in term of number of pixels as well as total storage size.

In order to understand how currently available JPEG2000 viewers deal with digital slides (and JPEG2000 in general), we decided to accomplish a survey of available software that we tested for use in digital pathology.

## Methods

Candidate JPEG2000 viewers were identified by searching the Web using the Google search engine. Viewers explicitly mentioning JPEG2000 were chosen, while those clearly aimed at more generic use were excluded from the study. Search has been focused on viewers that can be run on Windows, to be considered as a sample of the total number of viewers. In addition, commercial viewers were tested only when a demo was freely available.

JPEG2000 generic capabilities have been tested by means of conformance files as defined in [2]. This involved testing for codestreams Profile 0 and Profile 1, and JPEG2000 files.

For evaluating digital microscopy capabilities, two digital slides were opened into the viewers, with different features. One digital slide had pixel matrix size less than 65535 pixels in both axes, the other was larger than 65535 pixels.

For those viewers passing the latter test, time needed for opening was recorded, and interface features were examined to understand if and how they could be used for digital microscopy. This included a preliminary analysis of commands related to magnification (i.e., zoom) and image panning. For the latter in particular, the availability of keyboard, wand tool, window bars has been recorded.

## Results

According to the above-mentioned methods, twelve JPEG2000 viewers were identified, to which, for sake of comparison, also a digital microscopy viewer has been added in the analysis [1]. The next section will detail the observations made.

### Selected viewers

The following viewers were selected: kdu_show (KakaduSoftware), JP2View (Mustek), Brava!DesktopIXL (InformativeGraphics), OpenEV (GeoInnovations), XnView (Pierre Gougelet), eFotoXpress Viewer (eFotoExpress), JP2view (OptimiData), ER Viewer (Earth Resource Mapping), IrfanView (Irfan Skiljan), TNTAtas (MicroImages), Vliv (Frederic Delhoume), Stardust Image Viewer (Stardust Software), JVSView (University of Tampere).

Most of the viewers have been developed for geographic imaging, which is perhaps the closest field to digital microscopy, for either image size and interaction needs.

### File tests

Files developed for conformance tests have been downloaded from the web site .

An attempt to open relevant files has been made with all the viewers. This step included codestreams Profile 0 and Profile 1 (.j2k files), and JPEG2000 files (.jp2 files). Furthermore, digital slides produced starting from images acquired with eSlide [3] have been converted into PPM format by means of LargeMontage (University of Tampere, Finland) and then to JPEG2000 by means of kdu_compress (Kakadu Software, New Zealand).

Table 1 shows the results. Where the value "some" appears, it means that some image has not been opened and/or some image has been opened but with visualization errors.

**Table 1:** File test results

***Software***	***Codestream***		***jp2***	***eSlide coded in jp2***	
	*Profile 0*	**Profile 1**		*< 65536 pixel*	*> 65536 pixel*

Kdu_show	yes	yes	yes	yes	yes
JPEG2000 JP2 view	no	no	some	no	no
Brava! Desktop IXL	no	no	yes	no	no
OpenEV	some	some	some	yes	yes
XnView	some	some	some	no	no
eFotoXpress Viewer	no	no	yes	no	no
JP2view	some	no	some	no	no
ER Viewer	yes	yes	yes	yes	yes
IrfanView	some	some	yes	no	no
TNTatlas	no	no	some	no	no
Vliv	no	no	no	no	no
Stardust Image Viewer	some	no	some	no	no
JVSview	no	no	yes	yes	yes

### Digital slide tests

Last tests evaluated how the viewers passing the above tests behave in terms of digital pathology. The smaller of the two tested digital slides was opened and the time needed was measured; in addition to that, the interface has been examined to find the most important features needed for digital microscopy, i.e., magnification and field navigation. Table 2 shows the results.

**Table 2:** Digital slide test results

***Software***	***Opening Time *** *(seconds)*	***Magnification***	***Field navigation***		
			*Keyboard*	*Window bars*	*Panning*
Kdu_show	*2*	yes	yes	yes	yes
OpenEV	9	yes	yes	yes	no
ER Viewer	2	yes	yes	yes	yes
JVSview	2	yes	yes	yes	yes

## Discussion and conclusion

About half the viewers were unable to open all conformance JPEG2000 files. Considering large JPEG2000 images like those representing digital slides, only 31% of viewers were able to open both of them, i.e., 4 viewers, of which one is specialised in digital microscopy. All those viewers provided for magnification and field navigation through keyboard and window bars; three out of four also a panning tool, which is really useful for microscopy.

It can be concluded that digital microscopy, even if based on a standard format as JPEG2000, at present cannot rely on standardised software, but specific digital microscopy viewers are to be developed to deal with the large images produced in this field.
